# The Influence of Sirtuin 6 on Chondrocyte Senescence in Osteoarthritis Under Aging: Focusing on Mitochondrial Dysfunction and Oxidative Stress

**DOI:** 10.3390/antiox14101228

**Published:** 2025-10-13

**Authors:** Huiying Zhao, Wei Wu

**Affiliations:** 1 School of Exercise and Health, Shanghai University of Sports, Shanghai 200438, China; zhaohy1237@163.com; 2 School of Athletic Performance, Shanghai University of Sports, Shanghai 200438, China

**Keywords:** oxidative stress, osteoarthritis, mitochondrial dysfunction, Sirtuin 6, aging

## Abstract

Osteoarthritis (OA) is one of the most common joint diseases worldwide, which is characterized by degenerative changes in articular cartilage and secondary osteophyte formation. Numerous factors influence OA, including aging, obesity, joint injury and chronic overloading. Among them, the senescence of chondrocytes is one of the key factors leading to OA. Chondrocyte senescence can trigger inflammatory responses, extracellular matrix (ECM) degradation, mitochondrial dysfunction and oxidative stress (OS), and autophagy. Sirtuin 6 (SIRT6), as a deacetylase related to aging, can regulate chondrocyte senescence and plays a certain part in OA. SIRT6 regulates the number and membrane integrity of mitochondria, alleviates excessive Reactive Oxygen Species (ROS) in mitochondria and reduces inflammation-mediated mitochondrial damage. In addition, SIRT6 can also regulate the activity of antioxidant enzymes, inhibit excessive ROS induced by inflammatory factors, and alleviate OS. However, as aging progresses, the activity of SIRT6 will decrease. Activating the activity of SIRT6 becomes a potential therapeutic target and has a certain alleviating effect on the development of OA. The supplementation of nicotinamide adenine dinucleotide (NAD+) precursors and SIRT6-specific activators can increase SIRT6, alleviate chondrocyte senescence, and reduce OA. This paper aims to focus on mitochondrial dysfunction and OS to explore SIRT6’s effects on OA chondrocytes’ senescence under aging and summarize the potential therapeutic targets for activating SIRT6 to provide assistance for the improvement of OA.

## 1. Introduction

Osteoarthritis (OA) is a common chronic degenerative joint disease, mainly involving articular cartilage, subchondral bone and synovium [1]. The clinical manifestations of OA mainly include limited joint range of motion, pain and stiffness [2]. Aging is a very important cause of OA. The occurrence of OA is constantly increasing with aging, causing great inconvenience to patients’ lives. Since 1990, the prevalence of OA worldwide has been on the rise. It is estimated that by 2050, nearly 1 billion people globally will suffer from OA [3]. The aging of chondrocytes can exacerbate OA and is a key contributor to OA. Chondrocyte senescence can trigger inflammatory responses, disordered extracellular matrix (ECM) degradation and matrix-degrading enzymes, mitochondrial dysfunction and oxidative stress (OS), and autophagy.

The sirtuins family proteins (SIRTs), as nicotinamide adenine dinucleotide (NAD+)-dependent deacetylases, play a significant role in regulating OS, metabolic homeostasis and DNA repair [4]. Sirtuin 6 (SIRT6), as a deacetylase related to aging, is located in the cell nucleus. SIRT6 is connected to mitochondrial dysfunction and OS. It exerts a strong influence on OA and has become a new focus in OA research [5]. SIRT6 can regulate the number and membrane integrity of mitochondria, alleviate the excessive generation of ROS, and further inhibit inflammatory response. In addition, SIRT6 can also regulate antioxidant enzymes, inhibit cell aging and the generation of related inflammation, and better protect mitochondrial function. However, as aging progresses, the activity of SIRT6 decreases [6]. Activating the activity of SIRT6 becomes a potential therapeutic target and may have a certain alleviating effect on the development of OA. Supplementing NAD+ precursors and SIRT6-specific activators can increase SIRT6, alleviate the aging of chondrocytes, and reduce OA.

In this review, we have examined the connection between chondrocyte senescence and OA. We have systematically outlined the detrimental effects of chondrocyte senescence on OA patients from four perspectives: inflammatory response, ECM degradation, mitochondrial dysfunction and OS, and autophagy. Additionally, we focused on mitochondrial dysfunction and OS, expounding on the influence of SIRT6 on these two aspects and the related regulation. Finally, we also summarized the potential treatment for activating SIRT6, aiming to improve chondrocyte senescence and thus reduce the damage caused by OA.

## 2. Chondrocyte Aging and OA

OA arises through a multifaceted and heterogeneous array of mechanisms. Chondrocyte senescence is a primary trigger of OA. Articular chondrocytes are a type of low-proliferative cell. After injury, although chondrocytes can self-repair, they are more prone to aging, thereby increasing the possibility of developing osteoarthritis [7]. As aging, the body undergoes a series of changes, which mainly include inflammatory response, extracellular matrix degradation, mitochondrial dysfunction and OS, and autophagy.

### 2.1. Inflammatory Response

OA is further intensified by the chronic, low-grade systemic inflammation that accompanies aging—commonly referred to as “inflammatory senescence” [8]. The senescence-associated secretory phenotype (SASP) refers to a complex set of bioactive factors secreted by cells undergoing senescence, mainly including inflammatory cytokines, matrix metalloproteinases (MMPs), growth factors, etc. [9].

During aging, pro-inflammatory factors diffuse locally into the joint, promoting cartilage matrix degradation and stimulating synovial cells, exacerbating OA [10]. Interleukin-1β (IL-1β) is secreted mainly by macrophages and is a key pro-inflammatory factor [11]. IL-1β induces chondrocytes to express inducible nitric oxide synthase (iNOS) and thus produce NO by activating the NF-κB signaling pathway [12]. NO is cytotoxic at high concentrations, damages chondrocytes, inhibits anabolism, and promotes inflammatory responses [13]. IL-1β induces phospholipase A2 (PLA2) activity, which in turn activates cyclooxygenase-2 (COX-2) and increases prostaglandin E2 (PGE2) synthesis [14]. PGE2 is an important inflammatory mediator that stimulates the activation and proliferation of inflammatory cells, accelerates cartilage matrix degradation, and exacerbates OA [15]. ECM is an important component of the cartilage matrix. MMPs, as key matrix-degrading enzymes, have their expression levels upregulated due to inflammatory factors, which further aggravates inflammation and is closely related to OA. IL-1β will activate NF-κB, induce MMPs, and inhibit ECM synthesis [16].

Type II collagen is one of the main components of the ECM and works with proteoglycans to maintain the chondrocyte structure [17]. SRY-box transcription factor 9 (Sox9) is a key transcription factor in chondrocytes that activates the transcription of cartilage-specific genes [18]. IL-1β will reduce the expression of type II collagen and Sox9 in chondrocytes while inducing the synthesis and release of A Disintegrin and Metalloproteinase with Thrombospondin motifs (ADAMTS), degrading proteoglycans, and further damaging the cartilage matrix, thereby exacerbating OA [19,20]. Interleukin-6 (IL-6) is a multifunctional cytokine which can regulate immune responses as well as promote the recruitment and activation of inflammatory cells [21]. IL-6 activates the JAK–STAT3 signaling pathway, driving inflammatory cell infiltration and synoviocyte proliferation, inducing joint inflammation and exacerbating OA [22]. Meanwhile, IL-6 also directly stimulates MMPs and ADAMTS, altering the metabolic balance of chondrocytes and culminating in cartilage destruction and joint impairment [23]. Interleukin-8 (IL-8) acts as an inflammatory chemotactic factor that is capable of attracting and activating neutrophils, thereby further exacerbating the inflammatory response [24]. Furthermore, IL-8 can also induce the hypertrophy and differentiation of chondrocytes, increasing MMPs release and thereby destroying the cartilage matrix [25]. Tumor necrosis factor-α (TNF-α), a pro-inflammatory factor, is tightly associated with the severity of OA, and it can induce various inflammation-related genes [26]. The continuous inflammatory response will trigger inflammation in the periosteum and bone marrow, resulting in the destruction and remodeling of bone as well as further aggravating pathological changes of the joint. Growth factors are a type of polypeptide substance. Among them, insulin-like growth factors (IGFs) and vascular endothelial growth factor (VEGF) can regulate chondrocyte metabolism and play an important part in OA [27]. IGF-1 stimulates proteoglycan and type II collagen synthesis, drives mesenchymal stem-cell differentiation into chondrocytes, and thereby amplifies chondrocyte proliferation [28]. There is also a related study indicating that IGF-2 can inhibit MMP-13 and promote cartilage matrix synthesis [29]. VEGF enhances the supply of oxygen and nutrients by promoting angiogenesis, and it improves the survival and metabolic adaptation of chondrocytes [30].

### 2.2. ECM Degradation

The ECM mainly consists of collagen, proteoglycans and elastin, which can preserve cartilage architecture and function, endow cartilage with biomechanical properties and provide mechanical support. The senescent chondrocytes will promote the secretion of SASP, driving MMP upregulation and excessive ECM degradation [31].

The activity of MMPs can affect the cartilage matrix. MMP-13 is one of the key enzymes, which can specifically degrade type II collagen in the ECM [32]. The degradation of type II collagen, as the main structural protein of articular cartilage, will lead to damage of the integrity of the cartilage matrix, causing cartilage to lose its elasticity and compressive strength [33]. Senescent chondrocytes further release an array of matrix-degrading enzymes, including MMP-1, MMP-3, MMP-8 and MMP-9, which can work together to further destroy the collagen fiber network in the cartilage matrix [34]. Proteoglycans, as an important component of cartilage matrix, can absorb water and maintain the elasticity and lubricity of cartilage [35]. The senescent chondrocytes will secrete ADAMTS, which degrades proteoglycans, resulting in a decrease in the hydration capacity of the cartilage matrix and making cartilage drier and more fragile [36].

The senescent chondrocytes release inflammatory signals by secreting extracellular vesicles (EVs), and they also release more inflammatory mediators, thereby exacerbating the inflammatory response in the joint [37]. These EVs not only carry inflammatory factors but also can carry molecules such as mRNA and miRNA, regulating the gene expression of the receptor cells [38]. The inflammatory factors in EVs can engage receptors on surrounding cells, inducing other cells to enter an inflammatory state, continuously exacerbating the inflammation and damage of the joints [39]. A study has found that miRNAs in EVs can target anti-inflammatory genes in chondrocytes, inhibiting their expression and thereby exacerbating the inflammatory response [40]. Meanwhile, the miRNAs and proteins transmitted by EVs can regulate the gene expression within the receptor cells, inducing these cells to increase MMPs, thereby accelerating ECM degradation [41]. The damage caused by the ECM not only compromises cartilage mechanical integrity, rendering it increasingly vulnerable to damage from mechanical stress, but also activates the signaling pathways within cartilage cells, leading to more MMPs and inflammatory factors [42]. The damage to the ECM will also lead to insufficient nutrient supply to chondrocytes, which will have an impact on the nutrient transportation and metabolism of chondrocytes [43]. Insufficient nutrition will further accelerate the aging and death of chondrocytes, accelerate articular cartilage degeneration, and aggravate OA [44]. With aging, many molecular pathways in the joints become dysregulated, which affects ECM homeostasis in the cartilage, leading to structural disruption and the subsequent deterioration of its biomechanical properties.

### 2.3. Mitochondrial Dysfunction and OS

#### 2.3.1. Chondrocyte Senescence and Mitochondrial Dysfunction

OA is closely related to chondrocytes senescence caused by mitochondrial dysfunction, and mitochondrial dysfunction often stems from the imbalance of calcium homeostasis induced by electrophysiological disorders [45,46]. In healthy cartilage, the oscillations mediated by the TRPV4 and Piezo1 channels of calcium ions are crucial for maintaining mitochondrial function, matrix synthesis, and mechanical conduction [47,48]. However, in the pathological state of OA, long-term mechanical load or inflammatory factors can disrupt this calcium homeostasis, leading to continuous calcium influx and causing mitochondrial calcium overload [49]. Calcium overload not only disrupts the mitochondrial membrane potential but also induces the excessive production of ROS, further exacerbating mitochondrial dysfunction [50]. The mitochondrial dysfunction induced by calcium homeostasis imbalance will lead to a decrease in mitochondrial quantity, damage to membrane integrity, and mitochondrial DNA (mtDNA) damage, resulting in a reduction in mitochondrial membrane potential (MMP), a decrease in ATP production, an increase in excessive ROS generation, and an upregulation of MMP13 expression [51,52]. Due to the lack of protective histones and effective DNA repair mechanisms, mtDNA is vulnerable to oxidative stress damage and is released into the cytoplasm, activating inflammatory pathways and exacerbating the damage to chondrocytes, thereby accelerating the development of OA [53]. It is worth noting that the calcium influx mediated by ion channels such as TRPV4 and Piezo1 can also directly affect the activity of dehydrogenases in the tricarboxylic acid cycle by regulating mitochondrial calcium uptake, thereby controlling the efficiency of adenosine triphosphate (ATP) production [54,55]. Moderate calcium oscillations help maintain the flexibility of mitochondrial metabolism, while calcium imbalance leads to mitochondrial calcium overload, inducing the uncoupling of oxidative phosphorylation and reducing ATP synthesis [56,57]. Reduced ATP production will affect the synthetic metabolism of cells, leading to a decrease in the synthesis and repair of ECM and exacerbating the damage to chondrocytes [58].

**Figure 1:**
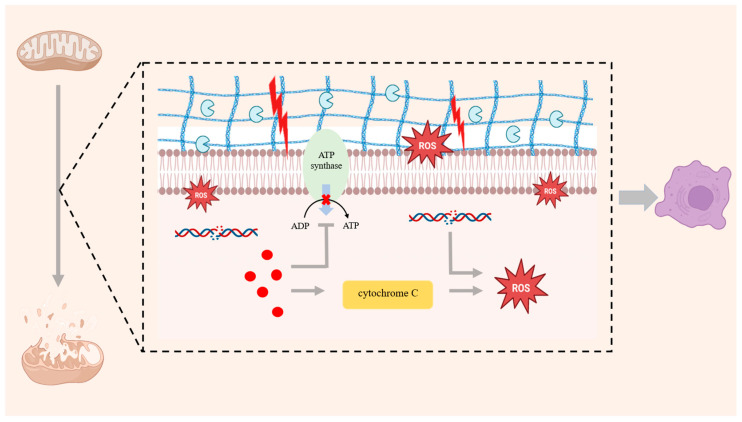
Mitochondrial dysfunction leads to reduced mitochondrial quantity and damage to membrane integrity. The excessive production of ROS not only damages mtDNA and the membrane structure but also damages intracellular proteins and lipids through OS, intensifying the inflammatory response. Inflammatory factors not only directly destroy chondrocytes but also interfere with the respiratory chain function of mitochondria, resulting in reduced ATP synthesis and exacerbating cartilage damage. Inflammatory mediators further drive cytochrome-c release from mitochondria and amplify intracellular ROS.

Mitochondrial dysfunction leads to the production of ROS, which not only damages the DNA and membrane structure of the mitochondria themselves but also causes damage to the proteins and lipids within the cells through OS [59]. Excessive ROS will activate NF-κB signaling pathway and upregulate MMP13 [60]. Meanwhile, mitochondrial dysfunction disrupts the balance between ECM synthesis and degradation, which also drives a marked increase in MMP13 expression. Elevated MMP-13 results in type II collagen and proteoglycans degradation, leading to cartilage matrix loss and cartilage degeneration, ultimately triggering OA [61]. The inflammatory factors produced in OA not only directly damage chondrocytes but also can further aggravate the damage to chondrocytes by influencing mitochondrial function [62,63]. Mitochondria generate the energy needed by cells through the oxidative phosphorylation process. Inflammatory factors interfere with the respiratory chain function of mitochondria, resulting in impaired oxidative phosphorylation and reduced ATP synthesis, thereby exacerbating cartilage damage [64]. A previous study has shown that TNF-α can suppress mitochondrial respiratory-chain complex activity, leading to a marked drop in ATP production [65]. Inflammatory factors can also induce excessive production of ROS in mitochondria, damaging the membrane structure, DNA and protein components of the mitochondria themselves [66]. Mitochondria are pivotal regulators of apoptosis. When mitochondria are damaged or have abnormal functions, they will release cytochrome C, which is a pro-apoptotic factor [67]. Inflammatory factors will stimulate mitochondria to release cytochrome C, thereby inducing the apoptosis of chondrocytes [68] (Figure 1).

#### 2.3.2. Chondrocyte Senescence and OS

OS denotes the overproduction of highly reactive molecules, primarily ROS and reactive nitrogen species (RNS), when the body encounters diverse injurious stimuli that disrupt redox homeostasis [69]. OS is intimately linked to an antioxidant system imbalance.

During OA, the senescence of chondrocytes is closely related to OS [70]. In chondrocytes, moderate OS can act as a signaling molecule to participate in the physiological regulation of the cells [71]. However, as aging occurs, OS will exceed the antioxidant capacity of the cells [72]. Excessive OS can interfere with various biomolecules within cells and disrupt their normal metabolic activities, leading to cellular dysfunction and even cell death [73]. Research reveals that OA cartilage exhibits diminished antioxidant enzyme activity, leading to ROS accumulation and a consequent amplification of OS [74]. The OS will continue to damage the DNA, proteins and lipids within the cells, leading to cellular dysfunction and apoptosis as well as further exacerbating mitochondrial dysfunction.

An excessive accumulation of ROS mainly induces chondrocyte apoptosis by PI3K/AKT and JNK signaling pathways, and it also reduces ECM synthesis [75]. The PI3K/AKT signaling pathway is pivotal for chondrocyte survival and ECM synthesis [76]. ROS directly inhibit the PI3K/AKT signal, resulting in a reduction in type II collagen and proteoglycans synthesis, and accelerating the apoptosis of chondrocytes. ROS reduce the phosphorylation levels of PI3K and AKT proteins through oxidative modification or inhibition, thereby inhibiting the survival signals of chondrocytes [77]. When the PI3K/AKT pathway is inhibited, the ability of chondrocytes to synthesize type II collagen and proteoglycans decreases, resulting in a reduction in ECM synthesis, destruction of the chondrocyte structure, and acceleration of their aging. In addition, ROS activate the JNK signaling pathway and the downstream NF-κB signaling pathway, induce pro-apoptotic proteins Bax and Bak, inhibit anti-apoptotic protein Bcl-2, increase the permeability of the outer mitochondrial membrane, release cytochrome C, and induce chondrocyte apoptosis, thereby promoting chondrocyte apoptosis [78]. The activated NF-κB pathway also induces a high expression of pro-inflammatory factors such as IL-1β and TNF-α, as well as MMPs, further accelerating the degradation of type II collagen and proteoglycans [79]. Ultimately, the reduction in chondrocyte synthesis, the increase in apoptosis, and the degradation of the matrix all contribute to the aging of chondrocytes and worsen OA.

**Figure 2:**
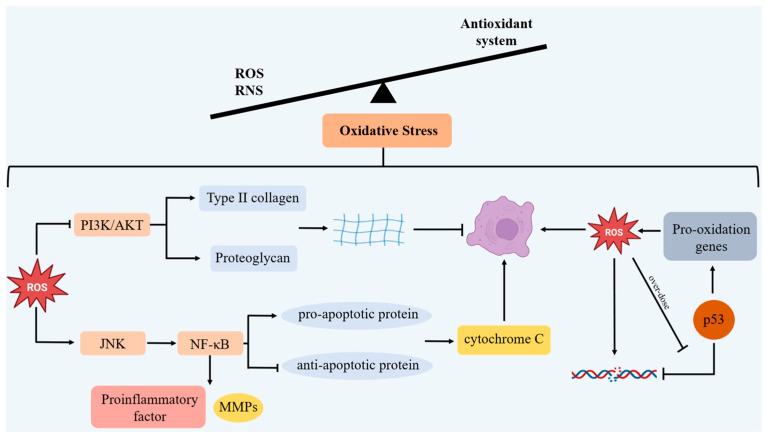
If there is excessive production of high-activity molecules ROS and RNS, and the antioxidant system becomes unbalanced, OS will be triggered. Excessive ROS will cause chondrocyte apoptosis through PI3K/AKT and JNK signaling pathways as well as reduce ECM synthesis. In addition, when ROS are overproduced, p53 will induce related pro-oxidative genes, accelerating chondrocyte senescence.

Excessive ROS damage to mtDNA causes mutations and instability in mtDNA. P53 is essential for safeguarding mtDNA integrity and stability. Under low ROS conditions, p53 promotes antioxidant genes to maintain homeostasis, activates DNA repair mechanism and regulates cell apoptosis to cope with mtDNA damage [80]. However, when ROS is excessively produced, p53 will induce related pro-oxidative genes. The products of these pro-oxidative genes will further increase ROS within the cells, exacerbating OS. Meanwhile, p53 also drives the transcription of pro-apoptotic genes. The products of these genes can promote ROS, thereby further exacerbating OS [81] (Figure 2).

### 2.4. Autophagy

The aging of chondrocytes is closely linked to autophagy. Autophagy is an internal degradation mechanism within cells that is used to remove damaged organelles and proteins as well as maintain intracellular homeostasis [82]. Autophagy, as a protective mechanism, can assist chondrocytes in coping with osteoarthritis and inflammatory responses, thus delaying the aging of chondrocytes [83]. So far, animal experiments have confirmed the significant role of autophagy in osteoarthritis. An experimental study indicated that the autophagic activity of cells in the knee joints of elderly mice was weakened, and apoptosis increased. Compared with young mice, the autophagosome count in the chondrocytes of elderly mice was significantly reduced, and the expression levels of autophagy-related proteins decreased. This was associated with an increase in apoptosis markers PARP and p85 in chondrocytes [84]. When autophagy occurs, the increase in LC3-II, Beclin-1 and Atg5 helps to maintain the quality and function of mitochondria, thereby protecting chondrocytes from damage caused by OS and inflammatory responses [85]. The active autophagic activity of cells is an important mechanism for maintaining the homeostasis of articular chondrocytes.

**Figure 3:**
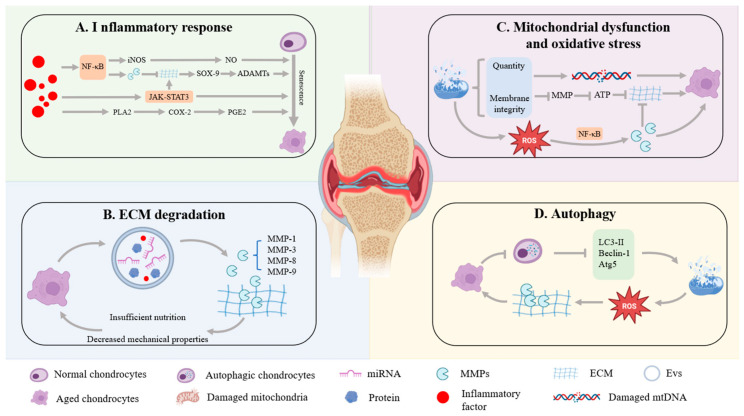
Inflammatory responses, ECM degradation, mitochondrial dysfunction and OS, as well as autophagy are the main causes of chondrocyte senescence. Inflammatory factors promote cartilage matrix degradation, aggravate inflammatory response, and accelerate the aging of chondrocytes. ECM degradation is also a major cause. The damaged ECM will affect the nutrient transportation and metabolism of chondrocytes, thereby exacerbating the aging and death of chondrocytes. Mitochondrial dysfunction and OS are very important causes. Mitochondrial dysfunction will affect the quantity and membrane integrity of mitochondria while concomitantly boosting ROS production, intensifying OS, and promoting chondrocytes senescence. An age-dependent decline in autophagy coincides with heightened chondrocyte apoptosis, accelerating their senescence.

As the body ages and cellular senescence intensifies, autophagy significantly decreases. LC3-II, Beclin-1 and Atg5 in chondrocytes decrease, disrupting intracellular homeostasis and hastening chondrocyte apoptosis along with cartilage matrix degradation [86]. The mitochondrial dysfunction and excessive accumulation of ROS caused by the reduced autophagy level will further aggravate OS, damage the DNA and proteins within the cells, and ultimately lead to cell apoptosis [87]. Dampened autophagy disrupts ECM turnover, upregulates MMP-13, and accelerates cartilage matrix breakdown [88]. The mammalian target of rapamycin (mTOR) protein in mammals serves as the primary negative regulator of autophagy. Its upregulation in OA inhibits the transmission of the autophagy signal and reduces the protective effect of autophagy on chondrocytes [89]. Rapamycin, as an inhibitor of the mTOR signaling pathway, can enhance autophagy regulatory factors and protect chondrocytes from damage [90]. An animal experiment has confirmed that injecting rapamycin into the joint cavity to promote autophagy in mouse chondrocytes can slow down OA [91]. Regulating autophagy levels is a potential strategy for treating OA, which helps reinstate chondrocyte functionality and slows down disease progression [92] (Figure 3).

## 3. SIRT6 and Mitochondrial Dysfunction

### 3.1. A Regulation of Number of Mitochondria and Integrity of the Mitochondrial Membranes

The number of mitochondria and the integrity of their membranes modulate mitochondrial function. Mitochondrial dysfunction accelerates chondrocyte senescence and exacerbates OA [93]. SIRT6 can regulate the related mitochondrial biosynthesis to maintain the quantity and membrane integrity of mitochondria. Peroxisome proliferator-activated receptor γ coactivator α (PGC-1α) governs mitochondrial biogenesis, and SIRT6 can activate PGC-1α expression [94]. Nuclear respiratory factor (NRF) is responsible for regulating the transcription of mitochondrial-related genes. Mitochondrial transcription factor A (TFAM) is involved in the replication and transcription of mtDNA. PGC-1α further activates NRF and TFAM, thereby enhancing mitochondrial biosynthesis [95]. In SIRT6-KO mice, the transcription and expression of mtDNA and PGC-1α genes decreased, resulting in reduced mitochondrial biosynthesis, insufficient ATP production, and mitochondrial dysfunction [96]. SIRT6 overexpression also activates the AMPK signaling pathway, regulates mitochondrial autophagy, removes damaged mitochondria, increases MMPs, and maintains mitochondrial function [97]. SIRT6 can also promote DNA repair and protect telomere function by means of deacetylation, thereby reducing the aging of chondrocytes [98]. Sirtuin 3 (SIRT3) and Sirtuin 4 (SIRT4) are members of the sirtuins family. SIRT6 can directly maintain the transcriptional levels of SIRT3 and SIRT4 [99]. In SIRT6-KO mice, SIRT3 and SIRT4 were significantly downregulated, leading to MMP disintegration and causing mitochondrial dysfunction. An exogenous supplementation of SIRT3 and SIRT4 could restore MMPs and maintain normal mitochondrial function, which also underscores the pivotal role of SIRT6 in this process [100]. The transcription factor Yin Yang 1 (YY1) is linked to the regulation of genes associated with skeletal muscle mitochondria and serves as the main switch for the expression of mitochondrial genes in skeletal muscle [101]. YY1 can recruit SIRT6 to the TFAM gene, causing H3K9 deacetylation, inhibiting the transcription of TFAM, and maintaining the synthesis of mitochondrial biology. When SIRT6 is absent, YY1 will cause a blockage in the input of mitochondrial proteins, ultimately resulting in a reduction in mitochondrial content and dysfunction, giving rise to the aging of chondrocytes and exacerbating OA [102].

### 3.2. A Regulation of ROS in Mitochondria

mtDNA damage is one of the important factors triggering mitochondrial dysfunction. Due to the absence of histone shielding around mtDNA and its relatively limited self-repair mechanism, it is more susceptible to damage from ROS [103]. ROS can lead to an exacerbation of oxidative damage to mtDNA, causing DNA strand breaks. This will affect the replication and transcription of mtDNA, thereby further aggravating the dysfunction of mitochondria [104]. Damaged mitochondria will lead to more ROS production, which in turn further damages mtDNA, forming a vicious cycle. SIRT6 has a positive regulatory effect on mtDNA transcription and mitochondrial biogenesis, and it can alleviate the damage caused by ROS as well as the excessive generation of ROS. An existing experimental study indicated that SIRT6 deficiency lowers mtDNA-encoded respiratory chain subunit transcripts and diminishes the total mitochondrial mass [100]. SIRT6 activates AMPK and enhances PGC-1α. At the same time, it can increase SOD2 through the AMPK–FOXO3a signaling axis and reduce mitochondrial ROS [105]. Transcription factor EB (TFEB) can regulate the fusion of autophagosomes and lysosomes, inhibit the aging and apoptosis of chondrocytes, and delay OA [106]. SIRT6 activates TFEB through deacetylation, promoting the formation of lysosomal mitochondrial autophagy, thereby promptly eliminating damaged mitochondria with excessive ROS levels and reducing the damage caused by ROS production [107]. In the OA cartilage samples from human bodies, the acetylation level of p66Shc significantly increased with the elevation of the Kellgren–Lawrence grading, and it exhibited an inverse correlation with SIRT6 expression [108]. The absence of SIRT6 will elevate p66Shc acetylation, causing an increase in mitochondrial ROS and the apoptosis of chondrocytes [109]. SIRT3 deficiency suppresses respiratory-chain subunit expression, curbs ATP generation, and disrupts ROS homeostasis. Meanwhile, the SIRT6–SIRT3 axis can provide certain antioxidant protection in OA chondrocytes [110]. SIRT6 can regulate SIRT3, catalyze the I subunit of the complex NDUFA9 and the II subunit of the complex SDHA, activate Superoxide Dismutase 2 (SOD2), and indirectly enhance the efficiency of the respiratory chain and the clearance ability of ROS [111].

### 3.3. A Regulation of Inflammation-Mediated Mitochondrial Damage

**Figure 4:**
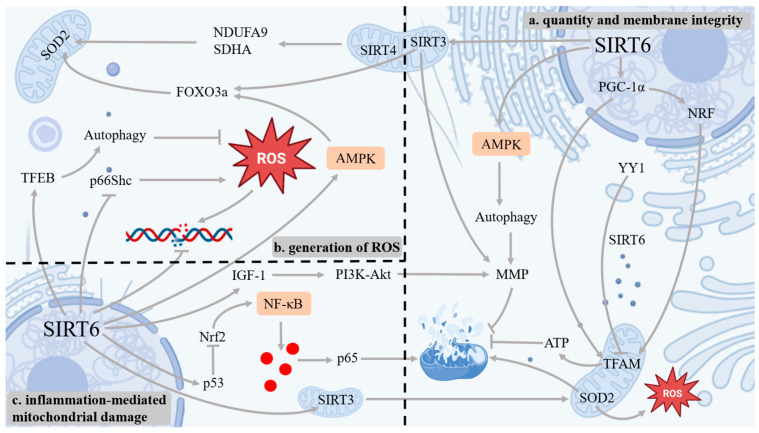
SIRT6 can regulate mitochondrial dysfunction. SIRT6 regulates mitochondrial biosynthesis related to PGC-1α to maintain the quantity and membrane integrity of mitochondria; SIRT6 can directly regulate SIRT3 and SIRT4, activate the AMPK signaling pathway to maintain MMP and reduce mitochondrial dysfunction. Activation of SIRT6 can enhance the expression of SOD2; SIRT6 activates TFEB, inhibits the increase in p66Shc acetylation, and reduces ROS. SIRT6 can inhibit inflammatory responses and reduce mitochondrial damage mediated by inflammation; SIRT6 can promote IGF-1 and its downstream PI3K–Akt signaling pathways as well as prevent the decrease in MMPs; SIRT6 upregulates SIRT3, increases the expression of SOD2, and maintains mitochondrial function.

Inflammatory factors can directly cause damage to chondrocytes, and at the same time, inflammatory responses can also damage mitochondria through multiple mechanisms, leading to mitochondrial dysfunction. SIRT6 can inhibit inflammatory factors to reduce mitochondrial damage. The stability and activity of Nuclear Factor erythroid 2-Related Factor 2 (Nrf2) are regulated by SIRT6, and the stability of Nrf2 is conducive to maintaining mitochondrial function. SIRT6 can reduce mitochondrial dysfunction caused by inflammatory factors by regulating the activity of Nrf2, thereby protecting chondrocytes [112]. Xia et al. pointed out that the activation of Sirt6 and Nrf2 signaling pathways can inhibit the NF-κB signaling pathway, which can significantly improve cartilage cell damage and prevent the aggravation of OA inflammation [113]. The acetylation of p53 induced by inflammatory factors will exacerbate mitochondrial apoptosis. SIRT6, by deacetylating p53 and promoting its ubiquitination degradation, blocks the inflammation-induced mitochondrial apoptosis signal and delays chondrocyte senescence [114]. The overexpression of SIRT6 can also inhibit the cellular senescence and NF-κB-mediated inflammatory response during OA, block the p65 subunit, and alleviate mitochondrial damage [115]. Similarly, when SIRT6 upregulates SIRT3, it causes the deacetylation of Forkhead box protein O3 (FOXO3a), activating antioxidant genes such as SOD2, reducing inflammatory stimulation, and maintaining mitochondrial function [99]. In addition, a study has shown that the absence of SIRT6 significantly downregulates IGF-1 and its downstream PI3K–Akt signaling pathway. The normal level of SIRT6 can maintain the metabolic imbalance of chondrocytes and prevent the decline of mitochondrial MMP, and it can also alleviate mitochondrial damage mediated by inflammation [116] (Figure 4).

## 4. SRIT6 and OS

### 4.1. A Regulation of Antioxidant Enzymes

SIRT6, as a deacetylase, provides a crucial pathway for regulating the antioxidant capacity of chondrocytes and can alleviate the damage caused by OS to chondrocytes [117]. Nrf2 mainly regulates antioxidant responses and cellular stress responses within the cell [118]. SIRT6 can activate Nrf2, thereby stimulating the production of more antioxidant enzymes and enhancing the cell’s antioxidant capacity [119]. Antioxidant enzymes play a crucial role in cells by removing excessive ROS and maintaining the redox balance within the cells. SIRT6 mainly regulates the activity of antioxidant enzymes by removing acetyl groups from these enzyme proteins, thereby alleviating OS. Heme Oxygenase-1 (HO-1) is a stress-induced enzyme that can produce metabolites with antioxidant and anti-inflammatory effects [120]. The metabolites of HO-1, such as bilirubin and carbon monoxide, can effectively eliminate excessive ROS and alleviate OS. The dysfunction of Keap1 is associated with various diseases, and mutations or abnormal expressions of Keap1 cause an abnormal activation of Nrf2. Currently, the research conducted by Mao et al. has demonstrated that SIRT6 can activate the Keap1/Nrf2/HO-1 signaling pathway, upregulating antioxidant enzymes and preventing the excessive production of ROS [121]. This antioxidant effect helps alleviate the cell damage caused by mitochondrial dysfunction, slowing down OA. SIRT6 overexpression can significantly increase Nrf2 and HO-1 while reducing Keap1. In IL-1β-induced chondrocytes, Sirt6 overexpression promotes DNA damage repair and inhibits chondrocyte senescence.

### 4.2. Inhibiting Excessive ROS Induced by Inflammatory Factors

**Figure 5:**
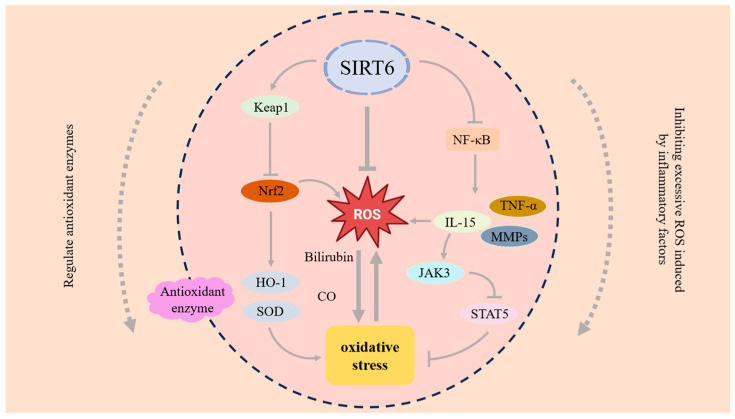
SRIT6 can regulate OS by modulating antioxidant enzymes and inhibiting the excessive ROS induced by inflammatory factors. SIRT6 can activate the Keap1/Nrf2/HO-1 signaling pathway, upregulate antioxidant enzymes, and reduce OS. In addition, SIRT6 regulates the NF-κB signaling pathway, inhibits related inflammatory factors and the IL-15/JAK3/STAT5 signaling pathway, and reduces OS.

Inflammatory factors will induce more ROS production, which will cause damage to chondrocytes and aggravate OA. SIRT6 can inhibit the inflammatory response and reduce the excessive ROS. SIRT6 regulates the NF-κB signaling pathway through deacetylation, inhibits related inflammatory factors and the activity of MMPs, prevents excessive ROS generation, and protects chondrocytes [115]. The animal experiments conducted by Jiang et al. showed that SIRT6 can regulate TNF-α through deacetylation, thereby inhibiting the inflammatory response [122]. Interleukin-15 (IL-15) is a pro-inflammatory cytokine. It can enhance the inflammatory response by activating the JAK3/STAT5 signaling pathway [123]. Signal Transducer and Activator of Transcription 5 (STAT5) is a type of signal transduction and transcriptional activation factor, belonging to the STAT protein family [124]. Upon IL-15–receptor engagement, IL-15 activates the Janus Kinase 3 (JAK3) gene, which in turn phosphorylates STAT5, resulting in increased various inflammatory factors [125]. The experiments conducted by Ji et al. demonstrated that SIRT6 can deacetylate the K163 site of STAT5, thereby inhibiting the phosphorylation and nuclear translocation of STAT5, reducing its transcriptional activity, and thereby suppressing the activation of the IL-15/JAK3/STAT5 signaling pathway and alleviating inflammatory response [126]. Suppressing this signaling axis is pivotal for mitigating chondrocyte senescence and inflammatory responses (Figure 5).

## 5. Potential Targeted Therapy of SIRT6

**Figure 6:**
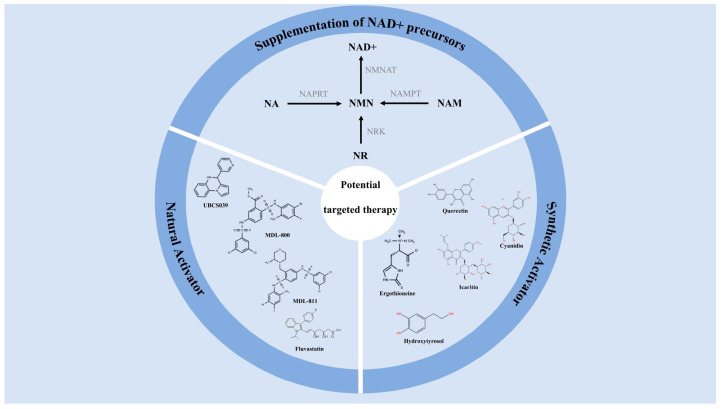
The potential targeted SIRT6 therapy can be mainly divided into three aspects: NAD+ precursor supplementation, natural activators of SIRT6, and synthetic activators of SIRT6. SIRT6 is dependent on NAD+. Supplementing NAD+ precursors can serve as a means to activate SIRT6. Natural activators of SIRT6 and synthetic activators of SIRT6 can activate SIRT6 through different mechanisms and pathways, reducing the aging of chondrocytes and slowing down the progression of OA.

SIRT6 expression in the chondrocytes of OA patients dropped markedly. This not only affected the normal functions of the chondrocytes but also exacerbated OA [127]. When SIRT6 expression is reduced, chondrocytes accelerate their aging process while the level of oxidative stress significantly increases, which will cause severe damage to the chondrocytes. A previous study has mentioned that the deficiency of SIRT6 will increase the severity of post-injury and age-related OA in the body, and it will aggravate the formation of cartilage degeneration, subchondral bone sclerosis and osteophytes [116]. Therefore, activating SIRT6 can alleviate the damage and aging of chondrocytes, making it a potential targeted therapy for OA [128] (Figure 6).

### 5.1. Supplementation of NAD+ Precursors

NAD+ is a necessary coenzyme that regulates metabolism, lifespan, DNA repair and the immune system. SIRT6 catalyzes reactions relying on NAD+ as a coenzyme. Age-dependent NAD+ decline curtails SIRT6 activity. Supplementing NAD+ precursors can increase the NAD+ levels in the body, thereby activating SIRT6, delaying the aging of chondrocytes and alleviating OA.

The supplementation of NAD+ precursors can restore NAD+ levels, activate SIRT6, promote DNA repair, mitochondrial function and cell regeneration, and delay the aging process. The main NAD+ precursors involved include nicotinamide riboside (NR), nicotinamide (NAM), nicotinic acid (NA), and nicotinamide mononucleotide (NMN). The metabolism of NR, NAM and NMN belongs to the salvage pathway. The metabolism of NA, however, belongs to the Preiss–Handler pathway. NR is a derivative of vitamin B3 and is mainly found in milk and dairy products [129]. NR ranks alongside NAM and NA as the third NAD+ vitamin precursor [130]. NR, through nicotinamide riboside kinases, such as NRK1 and NRK2, is phosphorylated to form NMN [131]. In study by Fang et al., it was mentioned that NRK1 and NRK2 can phosphorylate NR within the cell to form NMN. Subsequently, nicotinamide mononucleotide adenylyltransferase (NMNAT) catalyzes the generation of NAD+ through this pathway. This route is considered to be the most direct and efficient synthetic route in the NAD+ salvage pathway [132]. NAM is converted into NMN through NAMPT [133]. A study has confirmed that NAMPT is the rate-limiting enzyme in the NAD+ salvage pathway of mammalian cells, which can combine NAM with 5-phosphoribosyl-1-pyrophosphate (PRPP) to form NMN, thereby restoring NAD+ [134]. NA is converted into NMN through nicotinate phosphoribosyltransferase (NAPRT) [135]. When NMN increases NAD+, it can promote DNA breakage repair, slow down aging, and regulate metabolism [136]. NMN has been found to be present in milk, tomatoes, green beans, avocados and broccoli in relatively high amounts. It can also be supplemented through daily diet [137]. As of now, milk is one of the richest sources of NMN with a content of approximately 0.5–3.6 μM [138].

The main NAD+ precursor supplementations are detailed in Table 1.

### 5.2. Selective Activator of SIRT6

In recent years, studies have shown that some natural compounds and small molecule compounds can act as specific activators of SIRT6 and thus have potential therapeutic value for osteoarthritis. The specific activators of SIRT6 are mainly divided into natural activators and synthetic activators. Natural activators mainly include quercetin, cyanidin, ergothioneine, icaritin, and hydroxytyrosol. Synthetic activators mainly include UBCS039, MDL-800, MDL-811 and fluvastatin.

Quercetin, as a natural flavonoid compound, can activate SIRT6, regulate Nrf2, inhibit the NF-κB, and reduce pro-inflammatory cytokines such as IL-1β, TNF-α and IL-6 [139]. Quercetin can also inhibit JAK/STAT and reduce the production of subsequent inflammatory mediators [140]. Quercetin can also significantly reduce MMP-13 and COX-2, inhibit ECM degradation, and protect chondrocytes [141]. One study found that 20 µM quercetin was able to reduce MMP13 mRNA by 35–45% and COX-2 protein by 30%, but the level of relevant evidence remains relatively low [142]. In the rat OA model, the oral administration of 50 mg kg^−1^ of quercetin reduced the immunoreactive area of MMP-13 in cartilage by approximately 40%, and it decreased COX-2 by about 30% [143]. Quercetin is widely present in common fruits and vegetables such as onions, apples, broccoli, berries, green tea, red wine and cocoa, which can be consumed in daily meals [144]. Cyanidin is a natural anthocyanin compound that exhibits significant antioxidant and anti-aging properties [145]. Studies have shown cyanidin significantly increases the deacetylation activity of SIRT6, upregulates the expression of the FOXO3a gene, and simultaneously downregulates the expression of the Twist1 and GLUT1 genes [146]. This regulation of gene expression helps to reduce excessive ROS, thereby alleviating chondrocyte senescence. An in vitro experiment showed that cyanidin at a concentration of 460 µM could stimulate the deacetylation activity of SIRT6 by up to 55 times [147]. Cyanidin is widely present in blackberries, blackcurrants, grains, etc. Black rice and purple corn are the richest sources of cyanidin among cereal foods [148]. Ergothioneine possesses strong antioxidant properties, which can activate SIRT6 and regulate NF-κB, thereby further suppressing inflammatory responses [149]. Ergothioneine significantly reduces the breakdown of type II collagen and aggrecan in OA chondrocytes, and it inhibits various inflammatory factors [150]. In the 8-week DMM mouse OA model, when 50 mg/kg ergothioneine was administered intragastrically daily, the results showed that the Mankin score decreased by 32% and the area of subchondral bone sclerosis reduced by 28% [151]. Ergothioneine is mainly found in mushrooms, fermented soy products, fermented rice bran and spirulina. Mushrooms are usually the richest source of ergothioneine in human diet [152]. It is worth noting that due to the high ergothioneine content in certain plants, mushrooms, and spirulina, they have been utilized and their medicinal properties have been studied [153]. Icaritin, a secondary glycoside, is an active component extracted from the Chinese herb Herba Epimedii [154]. Icaritin alleviates inflammation through activating SIRT6 and suppressing NF-κB [155]. In the rabbit knee joint cartilage defect model, after 12 weeks of using epimedium glycosides, it was found that the expression of type II collagen was significantly increased, and the interface between the cartilage and the subchondral bone was well repaired [156]. Hydroxytyrosol is a phenolic compound mainly derived from olives that exhibits significant anti-inflammatory and antioxidant properties [157]. Hydroxytyrosol inhibits MMPs, thereby reducing inflammatory responses and cartilage degradation [158]. In an in vitro experiment, mouse knee joint cartilage was used for primary chondrocyte culture, and then IL-1β was stimulated and hydroxytyrosol was applied. Compared with the control group (set as 1.00, 0.85 ± 0.08) that was not stimulated by IL-1β, 50 µM of HT reduced the intensity of the MMP-13 band to 0.42 ± 0.05 (approximately 51% reduction) [159]. In daily diet, tea, olive oil and olives contain a certain amount of hydroxytyrosol, which can provide certain antioxidant protection for the body [160]. UBCS039 is the first artificially synthesized SIRT6 activator, which can significantly enhance SIRT6 [161]. Using UBCS039 for treatment can effectively inhibit NF-κB and inflammatory cytokines, and 40 µM UBCS039 (in vitro) or 20 mg kg^−1^ (in vivo) can reduce the main inflammatory indicators (IL-1β and TNF-α) by 55–62% [162]. UBCS039, by combining with SIRT6, can also increase ATP and prevent the aggravation of mitochondrial dysfunction [163]. MDL-800 is a common SIRT6 activator that can effectively alleviate the symptoms associated with OA [164]. The MDL-800 can improve OA by inhibiting inflammatory factors and promoting cartilage matrix synthesis. There was an experiment where MDL-800 was used to treat senescent chondrocytes. The results showed the chondrocytes treated with MDL-800 exhibited significantly lower DNA damage and effectively inhibited cell aging [165]. One week after the DMM mice surgery, MDL-800 was injected into the joint cavity. Eight weeks later, the samples were collected, showing that the Mankin score decreased by 2.1 points, the proportion of p16INK4a-positive chondrocytes decreased by 48%, and the fluorescence intensity of γH2AX decreased by 52% [126]. MDL-811 is a relatively new type of SIRT6 activator, and its activity is twice as high as that of MDL-800 [166]. An animal experiment has confirmed that when MDL-811 activates SIRT6, it can reduce TNF-α by 70% and IL-1β by 60%, effectively alleviating the inflammatory response [167]. Fluvastatin can bind to the acyl domain of SIRT6 substrates. Currently, it is also a broad-spectrum SIRT6 activator [168]. In mouse OA chondrocytes induced by IL-1β, fluvastatin can inhibit the activation of NF-κB and downregulate MMP-13 and ADAMTS [169]. One study showed that 10 µM fluvastatin significantly reduced the mortality rate of chondrocytes after trauma and downregulated MMPs (Table 2) [170].

Natural activators and synthetic activators of SIRT6 are detailed in Table 2.

Currently, the clinical data related to SIRT6 activators are limited, and they have not yet reached the stage of systematic clinical validation. There are no systematic clinical trials specifically evaluating the efficacy or safety of SIRT6 activators. Although SIRT6 activation has been demonstrated in animal models, its SIRT6 targeting has not been verified in humans, and there is a lack of clinical validation of SIRT6 as a biomarker.

## 6. Conclusions and Perspectives

OA is a chronic disease that affects middle-aged and elderly people. With aging, chondrocyte senescence has always been a key factor influencing OA. This review focuses on the factor of chondrocyte senescence and elaborates on the process by which chondrocyte senescence affects OA from the perspectives of inflammatory response, ECM degradation, mitochondrial dysfunction and OS, and autophagy. This review focuses primarily on mitochondrial dysfunction and OS, and it explores the relationship between chondrocyte senescence and these two factors.

Chondrocyte senescence can lead to mitochondrial dysfunction, decreasing mitochondrial quantity, which is damage to membrane integrity and mtDNA. Mitochondrial dysfunction leads to excessive ROS, triggering more inflammatory factors and exacerbating cartilage damage. OS has always been a key factor in chondrocyte senescence. Excessive ROS can trigger chondrocyte apoptosis through PI3K/AKT and JNK signaling pathways as well as reduce ECM synthesis. In addition, when ROS are excessively produced, p53 will induce the expression of related pro-oxidative genes, accelerating chondrocyte senescence. As a member of the SIRTs family, SIRT6 plays a certain part in regulating mitochondrial dysfunction and OS. SIRT6 can regulate the quantity and membrane integrity of mitochondria, inhibit excessive ROS production and regulate mitochondrial damage mediated by inflammation. In addition, SIRT6 can also regulate related antioxidant enzymes and inhibit excessive ROS induced by inflammatory factors. It is known that SIRT6 levels are markedly reduced in OA patients. Activating SIRT6 to alleviate chondrocyte senescence becomes a potential target for treating OA. Supplementing NAD+ precursors and using SIRT6-specific activators are two potential approaches. SIRT6-specific activators are divided into natural activators and synthetic activators. This review provides detailed discussions on both of them.

This review has summarized the relevant mechanisms by which SIRT6 regulates chondrocyte senescence and inhibits OA. Although activating SIRT6 shows positive effects in alleviating chondrocyte senescence, improving mitochondrial dysfunction and OS, its activation is not necessarily beneficial for chondrocytes in all situations, and there is currently a lack of systematic evidence for this. In addition, the natural activators of SIRT6 can be easily obtained from daily diet and are convenient to use, but there is no consensus on the “optimal effective dose”. On the other hand, although SIRT6-related activators have shown initial effectiveness in animal experiments, clinical verification is still lacking. As a deacetylase, SIRT6 has a wide range of substrates, including histones, transcription factors and metabolic enzymes, and excessive activation may interfere with the gene expression homeostasis or cause metabolic imbalance. The overexpression of SIRT6 has been found to have certain disadvantages in other cell types, but there is no evidence for it in chondrocytes. Therefore, future research needs to be further explored to provide more precise and safe strategies for OA intervention, avoiding the shift from protection to toxicity.

## Figures and Tables

**Table 1:** Main NAD+ precursor supplementation.

NAD+ Precursors	Metabolic Pathways	Mechanism	Application	Ref.
NR	Salvage pathway	Convert to NMN through NRK enzyme	Traditional NAD+ supplements	[115,116,117,118]
NAM	Salvage pathway	Convert to NMN through NAMPT		[119,120]
NA	Preiss–Handler pathway	Convert to NMN through NAPRT		[121]
NMN	Salvage pathway	Generate NAD+ through NMNAT	Repair DNA; Slow down aging; Regulate metabolism; Traditional NAD+ supplements	[122,123,124]

**Table 2:** Natural activators and synthetic activators of SIRT6.

Activators		Sources	Mechanisms	Evidence Level	Effects in OA Models	Ref.
Natural Activator	Quercetin	Onions, apples, broccoli, berries, green tea, red wine, cocoa, and vegetable juices	Regulate Nrf2; Reduce IL-1β, TNF-α and IL-6	Low	20 µM quercetin reduced MMP13 mRNA by 35–45% and COX-2 protein by 30%; Rat oral administration of 50 mg kg^−1^ of quercetin reduced the immunoreactive area of MMP-13 in cartilage by approximately 40%, and decreased COX-2 by about 30%	[139,140,141,142,143,144]
			Inhibit JAK/STAT; Reduce inflammatory mediators			
			Reduce MMP-13; Inhibit ECM degradation; Protect chondrocytes			
	Cyanidin	Blackberry, blackcurrant, black rice and purple corn	Upregulate FOXO3a; Downregulate Twist1 and GLUT1; Reduce excessive ROS production	Low	460 µM cyanidin can stimulate the deacetylation activity of SIRT6 by up to 55 times	[145,146,147,148]
	Ergothioneine	Mushrooms, fermented soy products, fermented rice bran and spirulina	Regulate NF-κB; Inhibit the inflammatory response	Medium	50 mg/kg ergothioneine decreased Mankin score by 32% and reduced the area of subchondral bone sclerosis by 28%	[149,150,151,152,153]
	Icaritin	Herba Epimedii	Regulate NF-κB; Exert anti-inflammatory effects	Low	In an animal experimental model, it was confirmed that the expression of type II collagen was upregulated and cartilage repair was significantly improved	[154,155,156]
	Hydroxytyrosol	Tea, olive oil and olives	Inhibit MMPs; Reduce inflammatory responses and cartilage degradation	Low	An in vitro mouse experiment showed 50 µM hydroxytyrosol led to a 51% reduction in MMP-13	[157,158,159,160]
			Eliminate free radicals; Promote autophagy; Prevent oxidative damage to chondrocytes			
Synthetic Activator	UBCS039	Artificially synthesized	Inhibit NF-κB; Reduce inflammatory factors	Low	40 µM UBCS039 (in vitro) or 20 mg kg^−1^ (in vivo) can reduce main inflammatory indicators (IL-1β and TNF-α) by 55–62%	[161,162,163]
			Increase ATP; Prevent aggravation of mitochondrial dysfunction			
	MDL-800		Inhibit inflammatory factors; Promote synthesis of cartilage matrix; Inhibit cell aging	Medium	MDL-800 resulted in a 2.1-point decrease in the Mankin score of DMM mice after 8 weeks, a 48% reduction in the proportion of p16INK4a-positive chondrocytes, and a 52% decrease in the fluorescence intensity of γH2AX	[126,164,165]
			Reduce DNA damage; Inhibit cell aging			
	MDL-811		Reduce TNF-α, IL-1β and IL-6; Alleviate inflammatory response	Low	An animal experiment showed that MDL-811 can reduce TNF-α by 70% and IL-1β by 60%	[166,167]
	Fluvastatin		Block NF-κB; Downregulate MMP-13 and ADAMTS	Low	10 µM fluvastatin reduced mortality rate of chondrocytes and downregulated MMPs	[168,169,170]

## Data Availability

No data were used for the research described in this review.

## Abbreviations

The following abbreviations are used in this manuscript:

OA	Osteoarthritis
SIRT6	Sirtuin6
ROS	Reactive Oxygen Species
NAD+	Nicotinamide adenine dinucleotide
SIRTs	Sirtuins
SASP	Senescence-associated secretory phenotype
MMPs	Matrix metalloproteinases
IL-1β	Interleukin—1β
iNOS	Inducible nitric oxide synthase
PLA2	Phospholipase A2
COX-2	Cyclooxygenase-2
PGE2	ProstaglandinE2
ECM	Extracellular matrix
Sox9	SRY-box transcription factor 9
ADAMTS	A Disintegrin and Metalloproteinase with Thrombospondin motifs
IL-6	Interleukin-6
IL-8	Interleukin-8
TNF-α	Tumor necrosis factor-α
IGF	Insulin-like growth factors
VEGF	Vascular endothelial growth factor
EVs	Extracellular vesicles
mtDNA	Mitochondrial DNA
MMP	Mitochondrial membrane potential
ATP	Adenosine triphosphate
OS	Oxidative stress
RNS	Reactive nitrogen species
mTOR	Mammalian target of rapamycin
PGC-1α	Peroxisome proliferators-activated receptor γ coactivator l alpha
NRF	Nuclear respiratory factor
TFAM	Mitochondrial transcription factor A
SIRT3	Sirtuin 3
SIRT4	Sirtuin 4
TFEB	Transcription factor EB
SOD2	Superoxide dismutase 2
Nrf2	Nuclear Factor erythroid 2-Related Factor 2
FOXO3a	Forkhead box protein O3
HO-1	Heme Oxygenase-1
IL-15	Interleukin-15
STAT5	Signal Transducer and Activator of Transcription 5
JAK3	Janus Kinase 3
NR	Nicotinamide riboside
NAM	Nicotinamide
NA	Nicotinic acid
NMN	Nicotinamide mononucleotide
NMNAT	Nicotinamide mononucleotide adenylyltransferase
PRPP	5-phosphoribosyl-1-pyrophosphate
NAPRT	Nicotinate phosphoribosyltransferase

## References

[1] Loeser R.F., Goldring S.R., Scanzello C.R., Goldring M.B. (2012). Osteoarthritis: A disease of the joint as an organ. Arthritis Rheum..

[2] Glyn-Jones S., Palmer A.J., Agricola R., Price A.J., Vincent T.L., Weinans H., Carr A.J. (2015). Osteoarthritis. Lancet.

[3] Chen X., Tang H., Lin J., Zeng R. (2023). Temporal trends in the disease burden of osteoarthritis from 1990 to 2019, and projections until 2030. PLoS ONE.

[4] Ji Z., Liu G.H., Qu J. (2025). Mitochondrial sirtuins, key regulators of aging. Life Med..

[5] Michishita E., McCord R.A., Berber E., Kioi M., Padilla-Nash H., Damian M., Cheung P., Kusumoto R., Kawahara T.L., Barrett J.C. (2008). SIRT6 is a histone H3 lysine 9 deacetylase that modulates telomeric chromatin. Nature.

[6] Liao C.Y., Kennedy B.K. (2016). SIRT6, oxidative stress, and aging. Cell Res..

[7] Ashraf S., Cha B.H., Kim J.S., Ahn J., Han I., Park H., Lee S.H. (2016). Regulation of senescence associated signaling mechanisms in chondrocytes for cartilage tissue regeneration. Osteoarthr. Cartil..

[8] Han Z., Wang K., Ding S., Zhang M. (2024). Cross-talk of inflammation and cellular senescence: A new insight into the occurrence and progression of osteoarthritis. Bone Res..

[9] Jacob J., Aggarwal A., Bhattacharyya S., Sahni D., Sharma V., Aggarwal A. (2025). Fisetin and resveratrol exhibit senotherapeutic effects and suppress cellular senescence in osteoarthritic cartilage-derived chondrogenic progenitor cells. Eur. J. Pharmacol..

[10] Coryell P.R., Diekman B.O., Loeser R.F. (2021). Mechanisms and therapeutic implications of cellular senescence in osteoarthritis. Nat. Rev. Rheumatol..

[11] Migliorini P., Italiani P., Pratesi F., Puxeddu I., Boraschi D. (2020). The IL-1 family cytokines and receptors in autoimmune diseases. Autoimmun. Rev..

[12] Brom M., Saxer F., Mindeholm L., Schieker M., Conaghan P.G. (2025). Is It Time to Revisit the Role of Interleukin-1 Inhibitors in Osteoarthritis?. Diabetes Metab. Syndr. Obes..

[13] Liu L., Luo P., Yang M., Wang J., Hou W., Xu P. (2022). The role of oxidative stress in the development of knee osteoarthritis: A comprehensive research review. Front. Mol. Biosci..

[14] Molnar V., Matišić V., Kodvanj I., Bjelica R., Jeleč Ž., Hudetz D., Rod E., Čukelj F., Vrdoljak T., Vidović D. (2021). Cytokines and Chemokines Involved in Osteoarthritis Pathogenesis. Int. J. Mol. Sci..

[15] Guan J., Chen C., Wu S., Zhu H. (2025). The Role of PGE2 in Age-related Diseases. Curr. Drug Targets.

[16] Jenei-Lanzl Z., Meurer A., Zaucke F. (2019). Interleukin-1β signaling in osteoarthritis–chondrocytes in focus. Cell. Signal..

[17] Yap A.X.W., You Y.X., Ajit Singh D.K., Mat S., Chong C.P., Mohamad Yahaya N.H., Maktar J.F., Abdul Rani R., Ooi T.C., Ismail M. (2025). Efficacy of oral nutrition supplementation enriched with hydroxymethylbutyrate (HMB) and undenatured type-II collagen (UC-II) combined with exercise training on osteoarthritis-related outcomes among adults with knee osteoarthritis in Klang Valley of Malaysia: Study protocol for a randomised controlled trial. BMJ Open.

[18] Cucchiarini M., Thurn T., Weimer A., Kohn D., Terwilliger E.F., Madry H. (2007). Restoration of the extracellular matrix in human osteoarthritic articular cartilage by overexpression of the transcription factor SOX9. Arthritis Rheum..

[19] Xu Z., Ke T., Zhang Y., Fu C., He W. (2020). Agonism of GPR120 prevented IL-1β-induced reduction of extracellular matrix through SOX-9. Aging.

[20] Tabeian H., Betti B.F., Dos Santos Cirqueira C., de Vries T.J., Lobbezoo F., Ter Linde A.V., Zandieh-Doulabi B., Koenders M.I., Everts V., Bakker A.D. (2019). IL-1β Damages Fibrocartilage and Upregulates MMP-13 Expression in Fibrochondrocytes in the Condyle of the Temporomandibular Joint. Int. J. Mol. Sci..

[21] Liao Y., Ren Y., Luo X., Mirando A.J., Long J.T., Leinroth A., Ji R.R., Hilton M.J. (2022). Interleukin-6 signaling mediates cartilage degradation and pain in posttraumatic osteoarthritis in a sex-specific manner. Sci. Signal..

[22] Huang B., Lang X., Li X. (2022). The role of IL-6/JAK2/STAT3 signaling pathway in cancers. Front. Oncol..

[23] Srirangan S., Choy E.H. (2010). The role of interleukin 6 in the pathophysiology of rheumatoid arthritis. Ther. Adv. Musculoskelet. Dis..

[24] Bernhard S., Hug S., Stratmann A.E.P., Erber M., Vidoni L., Knapp C.L., Thomaß B.D., Fauler M., Nilsson B., Nilsson Ekdahl K. (2021). Interleukin 8 Elicits Rapid Physiological Changes in Neutrophils That Are Altered by Inflammatory Conditions. J. Innate Immun..

[25] Cecil D.L., Rose D.M., Terkeltaub R., Liu-Bryan R. (2005). Role of interleukin-8 in PiT-1 expression and CXCR1-mediated inorganic phosphate uptake in chondrocytes. Arthritis Rheum..

[26] Baud V., Karin M. (2001). Signal transduction by tumor necrosis factor and its relatives. Trends Cell Biol..

[27] Tu P., Guo Y., Zheng S., Pan Y., Wang L., Ma Y. (2019). Research progress on signaling molecules involved in articular cartilage repair]. Sheng Wu Yi Xue Gong Cheng Xue Za Zhi.

[28] Mullen L.M., Best S.M., Ghose S., Wardale J., Rushton N., Cameron R.E. (2015). Bioactive IGF-1 release from collagen-GAG scaffold to enhance cartilage repair in vitro. J. Mater. Sci. Mater. Med..

[29] Uchimura T., Foote A.T., Smith E.L., Matzkin E.G., Zeng L. (2015). Insulin-Like Growth Factor II (IGF-II) Inhibits IL-1β-Induced Cartilage Matrix Loss and Promotes Cartilage Integrity in Experimental Osteoarthritis. J. Cell. Biochem..

[30] Hu K., Olsen B.R. (2017). Vascular endothelial growth factor control mechanisms in skeletal growth and repair. Dev. Dyn..

[31] Liu S., Deng Z., Chen K., Jian S., Zhou F., Yang Y., Fu Z., Xie H., Xiong J., Zhu W. (2022). Cartilage tissue engineering: From proinflammatory and anti-inflammatory cytokines to osteoarthritis treatments (Review). Mol. Med. Rep..

[32] Mueller M.B., Tuan R.S. (2011). Anabolic/Catabolic balance in pathogenesis of osteoarthritis: Identifying molecular targets. PM&R.

[33] Hu Q., Ecker M. (2021). Overview of MMP-13 as a Promising Target for the Treatment of Osteoarthritis. Int. J. Mol. Sci..

[34] Rowan A.D., Litherland G.J., Hui W., Milner J.M. (2008). Metalloproteases as potential therapeutic targets in arthritis treatment. Expert Opin. Ther. Targets.

[35] Fu P.J., Zheng S.Y., Luo Y., Ren Z.Q., Li Z.H., Wang Y.P., Lu B.B. (2025). Prg4 and Osteoarthritis: Functions, Regulatory Factors, and Treatment Strategies. Biomedicines.

[36] Nagase H., Kashiwagi M. (2003). Aggrecanases and cartilage matrix degradation. Arthritis Res. Ther..

[37] Li Z., Bi R., Zhu S. (2023). The Dual Role of Small Extracellular Vesicles in Joint Osteoarthritis: Their Global and Non-Coding Regulatory RNA Molecule-Based Pathogenic and Therapeutic Effects. Biomolecules.

[38] Miyaki S. (2018). Cartilage/chondrocyte research and osteoarthritis. The role of microRNAs and extracellular vesicles in osteoarthritis pathogenesis. Clin. Calcium.

[39] Zhou Y., Ming J., Li Y., Li B., Deng M., Ma Y., Chen Z., Zhang Y., Li J., Liu S. (2021). Exosomes derived from miR-126-3p-overexpressing synovial fibroblasts suppress chondrocyte inflammation and cartilage degradation in a rat model of osteoarthritis. Cell Death Discov..

[40] Jeon O.H., Wilson D.R., Clement C.C., Rathod S., Cherry C., Powell B., Lee Z., Khalil A.M., Green J.J., Campisi J. (2019). Senescence cell-associated extracellular vesicles serve as osteoarthritis disease and therapeutic markers. JCI Insight.

[41] Chen P., Zeng L., Wang T., He J., Xiong S., Chen G., Wang Q., Chen H., Xie J. (2025). The communication role of extracellular vesicles in the osteoarthritis microenvironment. Front. Immunol..

[42] Maldonado M., Nam J. (2013). The role of changes in extracellular matrix of cartilage in the presence of inflammation on the pathology of osteoarthritis. Biomed Res. Int..

[43] Yin H., Li M., Tian G., Ma Y., Ning C., Yan Z., Wu J., Ge Q., Sui X., Liu S. (2022). The role of extracellular vesicles in osteoarthritis treatment via microenvironment regulation. Biomater. Res..

[44] Sukhikh S., Noskova S., Ivanova S., Ulrikh E., Izgaryshev A., Babich O. (2021). Chondroprotection and Molecular Mechanism of Action of Phytonutraceuticals on Osteoarthritis. Molecules.

[45] Mobasheri A., Matta C., Giles W., Choi H., Ivanavicius S. (2024). The interplay between inflammatory mediators and mechanotransduction is mediated by ion channels in articular chondrocytes: Functional consequences in osteoarthritis. Phys. Life Rev..

[46] Duong V., Abdel Shaheed C., Ferreira M.L., Narayan S.W., Venkatesha V., Hunter D.J., Zhu J., Atukorala I., Kobayashi S., Goh S.L. (2025). Risk factors for the development of knee osteoarthritis across the lifespan: A systematic review and meta-analysis. Osteoarthr. Cartil..

[47] Servin-Vences M.R., Richardson J., Lewin G.R., Poole K. (2018). Mechanoelectrical transduction in chondrocytes. Clin. Exp. Pharmacol. Physiol..

[48] Gao W., Hasan H., Anderson D.E., Lee W. (2022). The Role of Mechanically-Activated Ion Channels Piezo1, Piezo2, and TRPV4 in Chondrocyte Mechanotransduction and Mechano-Therapeutics for Osteoarthritis. Front. Cell Dev. Biol..

[49] Zhang K., Wang L., Liu Z., Geng B., Teng Y., Liu X., Yi Q., Yu D., Chen X., Zhao D. (2021). Mechanosensory and mechanotransductive processes mediated by ion channels in articular chondrocytes: Potential therapeutic targets for osteoarthritis. Channels.

[50] Guan M., Han X., Liao B., Han W., Chen L., Zhang B., Peng X., Tian Y., Xiao G., Li X. (2025). LIPUS Promotes Calcium Oscillation and Enhances Calcium Dependent Autophagy of Chondrocytes to Alleviate Osteoarthritis. Adv. Sci..

[51] Matta C., Takács R., Ducza L., Ebeid R.A., Choi H., Mobasheri A. (2023). Ion channels involved in inflammation and pain in osteoarthritis and related musculoskeletal disorders. Am. J. Physiol. Cell Physiol..

[52] Tan S., Sun Y., Li S., Wu H., Ding Y. (2025). The impact of mitochondrial dysfunction on osteoarthritis cartilage: Current insights and emerging mitochondria-targeted therapies. Bone Res..

[53] Akhmedov A.T., Marín-García J. (2015). Mitochondrial DNA maintenance: An appraisal. Mol. Cell. Biochem..

[54] Mobasheri A., Rayman M.P., Gualillo O., Sellam J., van der Kraan P., Fearon U. (2017). The role of metabolism in the pathogenesis of osteoarthritis. Nat. Rev. Rheumatol..

[55] Saberi M., Zhang X., Mobasheri A. (2021). Targeting mitochondrial dysfunction with small molecules in intervertebral disc aging and degeneration. Geroscience.

[56] Staunton C.A., Lewis R., Barrett-Jolley R. (2013). Ion channels and osteoarthritic pain: Potential for novel analgesics. Curr. Pain Headache Rep..

[57] Wang X., Li X. (2023). Regulation of pain neurotransmitters and chondrocytes metabolism mediated by voltage-gated ion channels: A narrative review. Heliyon.

[58] Wang J., Li J., Song D., Ni J., Ding M., Huang J., Yan M. (2020). AMPK: Implications in osteoarthritis and therapeutic targets. Am. J. Transl. Res..

[59] Minguzzi M., Cetrullo S., D’Adamo S., Silvestri Y., Flamigni F., Borzì R.M. (2018). Emerging Players at the Intersection of Chondrocyte Loss of Maturational Arrest, Oxidative Stress, Senescence and Low-Grade Inflammation in Osteoarthritis. Oxidative Med. Cell Longev..

[60] Choi M.C., Jo J., Park J., Kang H.K., Park Y. (2019). NF-κB Signaling Pathways in Osteoarthritic Cartilage Destruction. Cells.

[61] Otero M., Plumb D.A., Tsuchimochi K., Dragomir C.L., Hashimoto K., Peng H., Olivotto E., Bevilacqua M., Tan L., Yang Z. (2012). E74-like factor 3 (ELF3) impacts on matrix metalloproteinase 13 (MMP13) transcriptional control in articular chondrocytes under proinflammatory stress. J. Biol. Chem..

[62] Qi Z., Zhu J., Cai W., Lou C., Li Z. (2024). The role and intervention of mitochondrial metabolism in osteoarthritis. Mol. Cell. Biochem..

[63] Sanchez-Lopez E., Coras R., Torres A., Lane N.E., Guma M. (2022). Synovial inflammation in osteoarthritis progression. Nat. Rev. Rheumatol..

[64] Shen X., Hu J., Wang C., Wang H., Zhong H., Zhang Z., Wen G., Wang L., Dong M., Tian Y. (2025). Mitochondria-targeted NAD^+^/O_2_ co-delivery interpenetrating network hydrogel for respiratory chain restoration and osteoarthritis therapy. J. Control. Release.

[65] Ferroni L., Zago M., Patergnani S., Campbell S.E., Hébert L., Nielsen M., Scarpa C., Bassetto F., Pinton P., Zavan B. (2020). Fluorescent Light Energy (FLE) Acts on Mitochondrial Physiology Improving Wound Healing. J. Clin. Med..

[66] Lyu Y., Wang T., Huang S., Zhang Z. (2023). Mitochondrial Damage-Associated Molecular Patterns and Metabolism in the Regulation of Innate Immunity. J. Innate Immun..

[67] Wang C., Youle R.J. (2009). The role of mitochondria in apoptosis*. Annu. Rev. Genet..

[68] Lood C., Blanco L.P., Purmalek M.M., Carmona-Rivera C., De Ravin S.S., Smith C.K., Malech H.L., Ledbetter J.A., Elkon K.B., Kaplan M.J. (2016). Neutrophil extracellular traps enriched in oxidized mitochondrial DNA are interferogenic and contribute to lupus-like disease. Nat. Med..

[69] Ansari M.Y., Ahmad N., Haqqi T.M. (2020). Oxidative stress and inflammation in osteoarthritis pathogenesis: Role of polyphenols. Biomed. Pharmacother..

[70] Yudoh K., van Trieu N., Nakamura H., Hongo-Masuko K., Kato T., Nishioka K. (2005). Potential involvement of oxidative stress in cartilage senescence and development of osteoarthritis: Oxidative stress induces chondrocyte telomere instability and downregulation of chondrocyte function. Arthritis Res. Ther..

[71] Kan S., Duan M., Liu Y., Wang C., Xie J. (2021). Role of Mitochondria in Physiology of Chondrocytes and Diseases of Osteoarthritis and Rheumatoid Arthritis. Cartilage.

[72] Lepetsos P., Papavassiliou A.G. (2016). ROS/oxidative stress signaling in osteoarthritis. Biochim. Biophys. Acta.

[73] Ramasamy T.S., Yee Y.M., Khan I.M. (2021). Chondrocyte Aging: The Molecular Determinants and Therapeutic Opportunities. Front. Cell Dev. Biol..

[74] Huang K., Cai H. (2025). The role of chondrocyte senescence in osteoarthritis pathogenesis and therapeutic implications. Exp. Gerontol..

[75] Zahan O.M., Serban O., Gherman C., Fodor D. (2020). The evaluation of oxidative stress in osteoarthritis. Med. Pharm. Rep..

[76] Chen N.Y., Lu K., Yuan J.M., Li X.J., Gu Z.Y., Pan C.X., Mo D.L., Su G.F. (2021). 3-Arylamino-quinoxaline-2-carboxamides inhibit the PI3K/Akt/mTOR signaling pathways to activate P53 and induce apoptosis. Bioorganic Chem..

[77] Xu J., Yi Y., Li L., Zhang W., Wang J. (2015). Osteopontin induces vascular endothelial growth factor expression in articular cartilage through PI3K/AKT and ERK1/2 signaling. Mol. Med. Rep..

[78] Campbell K.J., Tait S.W.G. (2018). Targeting BCL-2 regulated apoptosis in cancer. Open Biol..

[79] Zhang G.Z., Liu M.Q., Chen H.W., Wu Z.L., Gao Y.C., Ma Z.J., He X.G., Kang X.W. (2021). NF-κB signalling pathways in nucleus pulposus cell function and intervertebral disc degeneration. Cell Prolif..

[80] Cheresh P., Kim S.J., Tulasiram S., Kamp D.W. (2013). Oxidative stress and pulmonary fibrosis. Biochim. Biophys. Acta.

[81] Liu B., Chen Y., St Clair D.K. (2008). ROS and p53: A versatile partnership. Free Radic. Biol. Med..

[82] Duan R., Xie H., Liu Z.Z. (2020). The Role of Autophagy in Osteoarthritis. Front. Cell Dev. Biol..

[83] Mizushima N., Komatsu M. (2011). Autophagy: Renovation of cells and tissues. Cell.

[84] Caramés B., Olmer M., Kiosses W.B., Lotz M.K. (2015). The relationship of autophagy defects to cartilage damage during joint aging in a mouse model. Arthritis Rheumatol..

[85] Hill S.M., Wrobel L., Rubinsztein D.C. (2019). Correction to: Post-translational modifications of Beclin 1 provide multiple strategies for autophagy regulation. Cell Death Differ..

[86] Liu Z., Wang T., Sun X., Nie M. (2023). Autophagy and apoptosis: Regulatory factors of chondrocyte phenotype transition in osteoarthritis. Hum. Cell.

[87] Andrei C., Mihai D.P., Nitulescu G.M., Nitulescu G., Zanfirescu A. (2024). Modulating Autophagy in Osteoarthritis: Exploring Emerging Therapeutic Drug Targets. Int. J. Mol. Sci..

[88] Huang W., Ao P., Li J., Wu T., Xu L., Deng Z., Chen W., Yin C., Cheng X. (2017). Autophagy Protects Advanced Glycation End Product-Induced Apoptosis and Expression of MMP-3 and MMP-13 in Rat Chondrocytes. Biomed Res. Int..

[89] Ryan P.J., Uranga S., Stanelle S.T., Lewis M.H., O’Reilly C.L., Cardin J.M., Deaver J.W., Morton A.B., Fluckey J.D. (2024). The autophagy inhibitor NSC185058 suppresses mTORC1-mediated protein anabolism in cultured skeletal muscle. Sci. Rep..

[90] Caramés B., Hasegawa A., Taniguchi N., Miyaki S., Blanco F.J., Lotz M. (2012). Autophagy activation by rapamycin reduces severity of experimental osteoarthritis. Ann. Rheum. Dis..

[91] Cejka D., Hayer S., Niederreiter B., Sieghart W., Fuereder T., Zwerina J., Schett G. (2010). Mammalian target of rapamycin signaling is crucial for joint destruction in experimental arthritis and is activated in osteoclasts from patients with rheumatoid arthritis. Arthritis Rheum..

[92] Li Y.S., Zhang F.J., Zeng C., Luo W., Xiao W.F., Gao S.G., Lei G.H. (2016). Autophagy in osteoarthritis. Jt. Bone Spine.

[93] Shao Y., Zhang H., Guan H., Wu C., Qi W., Yang L., Yin J., Zhang H., Liu L., Lu Y. (2024). PDZK1 protects against mechanical overload-induced chondrocyte senescence and osteoarthritis by targeting mitochondrial function. Bone Res..

[94] Zhang T., Wang L., Duan X., Niu Y., Li M., Yun L., Sun H., Ma Y., Guo Y. (2023). Sirtuins mediate mitochondrial quality control mechanisms: A novel therapeutic target for osteoporosis. Front. Endocrinol..

[95] Finck B.N., Kelly D.P. (2006). PGC-1 coactivators: Inducible regulators of energy metabolism in health and disease. J. Clin. Investig..

[96] Song M.Y., Han C.Y., Moon Y.J., Lee J.H., Bae E.J., Park B.H. (2022). Sirt6 reprograms myofibers to oxidative type through CREB-dependent Sox6 suppression. Nat. Commun..

[97] Fan Y., Yang Q., Yang Y., Gao Z., Ma Y., Zhang L., Liang W., Ding G. (2019). Sirt6 Suppresses High Glucose-Induced Mitochondrial Dysfunction and Apoptosis in Podocytes through AMPK Activation. Int. J. Biol. Sci..

[98] Nagai K., Matsushita T., Matsuzaki T., Takayama K., Matsumoto T., Kuroda R., Kurosaka M. (2015). Depletion of SIRT6 causes cellular senescence, DNA damage, and telomere dysfunction in human chondrocytes. Osteoarthr. Cartil..

[99] Wei W., Li T., Chen J., Fan Z., Gao F., Yu Z., Jiang Y. (2024). SIRT3/6: An amazing challenge and opportunity in the fight against fibrosis and aging. Cell. Mol. Life Sci..

[100] Smirnov D., Eremenko E., Stein D., Kaluski S., Jasinska W., Cosentino C., Martinez-Pastor B., Brotman Y., Mostoslavsky R., Khrameeva E. (2023). SIRT6 is a key regulator of mitochondrial function in the brain. Cell Death Dis..

[101] Gordon S., Akopyan G., Garban H., Bonavida B. (2006). Transcription factor YY1: Structure, function, and therapeutic implications in cancer biology. Oncogene.

[102] Kawamura K., Higuchi T., Fujiwara S. (2021). YAF2-Mediated YY1-Sirtuin6 Interactions Responsible for Mitochondrial Downregulation in Aging Tunicates. Mol. Cell. Biol..

[103] Zhang Z., Huang Q., Zhao D., Lian F., Li X., Qi W. (2023). The impact of oxidative stress-induced mitochondrial dysfunction on diabetic microvascular complications. Front. Endocrinol..

[104] Zhao M., Wang Y., Li L., Liu S., Wang C., Yuan Y., Yang G., Chen Y., Cheng J., Lu Y. (2021). Mitochondrial ROS promote mitochondrial dysfunction and inflammation in ischemic acute kidney injury by disrupting TFAM-mediated mtDNA maintenance. Theranostics.

[105] Li J., Yu D., Chen S., Liu Y., Shi J., Zhang J., Wen P., Wang Z., Li J., Guo W. (2020). Sirt6 opposes glycochenodeoxycholate-induced apoptosis of biliary epithelial cells through the AMPK/PGC-1α pathway. Cell Biosci..

[106] Zheng G., Zhan Y., Li X., Pan Z., Zheng F., Zhang Z., Zhou Y., Wu Y., Wang X., Gao W. (2018). TFEB, a potential therapeutic target for osteoarthritis via autophagy regulation. Cell Death Dis..

[107] Lu Y., Yang J., Wu Q., Wang X. (2025). The Role and Molecular Pathways of SIRT6 in Senescence and Age-related Diseases. Adv. Biol..

[108] Collins J.A., Kapustina M., Bolduc J.A., Pike J.F.W., Diekman B.O., Mix K., Chubinskaya S., Eroglu E., Michel T., Poole L.B. (2021). Sirtuin 6 (SIRT6) regulates redox homeostasis and signaling events in human articular chondrocytes. Free Radic. Biol. Med..

[109] Kanfi Y., Naiman S., Amir G., Peshti V., Zinman G., Nahum L., Bar-Joseph Z., Cohen H.Y. (2012). The sirtuin SIRT6 regulates lifespan in male mice. Nature.

[110] He Y., Wu Z., Xu L., Xu K., Chen Z., Ran J., Wu L. (2020). The role of SIRT3-mediated mitochondrial homeostasis in osteoarthritis. Cell. Mol. Life Sci..

[111] Pirinen E., Lo Sasso G., Auwerx J. (2012). Mitochondrial sirtuins and metabolic homeostasis. Best Pract. Res. Clin. Endocrinol. Metab..

[112] Pan H., Guan D., Liu X., Li J., Wang L., Wu J., Zhou J., Zhang W., Ren R., Zhang W. (2016). SIRT6 safeguards human mesenchymal stem cells from oxidative stress by coactivating NRF2. Cell Res..

[113] Xia W., Xiao J., Tong C., Lu J., Tu Y., Li S., Ni L., Shi Y., Luo P., Zhang X. (2023). Orientin inhibits inflammation in chondrocytes and attenuates osteoarthritis through Nrf2/NF-κB and SIRT6/NF-κB pathway. J. Orthop. Res..

[114] Wood M., Rymarchyk S., Zheng S., Cen Y. (2018). Trichostatin A inhibits deacetylation of histone H3 and p53 by SIRT6. Arch. Biochem. Biophys..

[115] Wu Y., Chen L., Wang Y., Li W., Lin Y., Yu D., Zhang L., Li F., Pan Z. (2015). Overexpression of Sirtuin 6 suppresses cellular senescence and NF-κB mediated inflammatory responses in osteoarthritis development. Sci. Rep..

[116] Collins J.A., Kim C.J., Coleman A., Little A., Perez M.M., Clarke E.J., Diekman B., Peffers M.J., Chubinskaya S., Tomlinson R.E. (2023). Cartilage-specific Sirt6 deficiency represses IGF-1 and enhances osteoarthritis severity in mice. Ann. Rheum. Dis..

[117] Chang A.R., Ferrer C.M., Mostoslavsky R. (2020). SIRT6, a Mammalian Deacylase with Multitasking Abilities. Physiol. Rev..

[118] Liu X., Ren S., Li Z., Hao D., Zhao X., Zhang Z., Liu D. (2023). Sirt6 mediates antioxidative functions by increasing Nrf2 abundance. Exp. Cell Res..

[119] Shi J., Chen L., Wang X., Ma X. (2025). SIRT6 inhibits endoplasmic reticulum stress-mediated ferroptosis by activating Nrf2/HO-1 signaling to alleviate osteoarthritis. Inflamm. Res..

[120] Cuadrado A., Rojo A.I., Wells G., Hayes J.D., Cousin S.P., Rumsey W.L., Attucks O.C., Franklin S., Levonen A.L., Kensler T.W. (2019). Therapeutic targeting of the NRF2 and KEAP1 partnership in chronic diseases. Nat. Rev. Drug Discov..

[121] Mao L.W., Jiang Q.Y., Meng N., Xiao L., Zhang Q., Chen Y.X., Liu L.J., Wang L. (2024). Sirt6 promotes DNA damage repair in osteoarthritis chondrocytes by activating the Keap1/Nrf2/HO-1 signaling pathway. Cell Cycle.

[122] Jiang H., Khan S., Wang Y., Charron G., He B., Sebastian C., Du J., Kim R., Ge E., Mostoslavsky R. (2013). SIRT6 regulates TNF-α secretion through hydrolysis of long-chain fatty acyl lysine. Nature.

[123] Krolopp J.E., Thornton S.M., Abbott M.J. (2016). IL-15 Activates the Jak3/STAT3 Signaling Pathway to Mediate Glucose Uptake in Skeletal Muscle Cells. Front. Physiol..

[124] Tóthová Z., Tomc J., Debeljak N., Solár P. (2021). STAT5 as a Key Protein of Erythropoietin Signalization. Int. J. Mol. Sci..

[125] Régnier P., Le Joncour A., Maciejewski-Duval A., Desbois A.C., Comarmond C., Rosenzwajg M., Klatzmann D., Cacoub P., Saadoun D. (2020). Targeting JAK/STAT pathway in Takayasu’s arteritis. Ann. Rheum. Dis..

[126] Ji M.L., Jiang H., Li Z., Geng R., Hu J.Z., Lin Y.C., Lu J. (2022). Sirt6 attenuates chondrocyte senescence and osteoarthritis progression. Nat. Commun..

[127] Sun K., Wu Y., Zeng Y., Xu J., Wu L., Li M., Shen B. (2022). The role of the sirtuin family in cartilage and osteoarthritis: Molecular mechanisms and therapeutic targets. Arthritis Res. Ther..

[128] Chen J., Chen S., Cai D., Wang Q., Qin J. (2022). The role of Sirt6 in osteoarthritis and its effect on macrophage polarization. Bioengineered.

[129] Makarov M.V., Migaud M.E. (2019). Syntheses and chemical properties of β-nicotinamide riboside and its analogues and derivatives. Beilstein J. Org. Chem..

[130] Bogan K.L., Brenner C. (2008). Nicotinic acid, nicotinamide, and nicotinamide riboside: A molecular evaluation of NAD^+^ precursor vitamins in human nutrition. Annu. Rev. Nutr..

[131] Yu P., Cai X., Liang Y., Wang M., Yang W. (2020). Roles of NAD^+^ and Its Metabolites Regulated Calcium Channels in Cancer. Molecules.

[132] Fang E.F., Lautrup S., Hou Y., Demarest T.G., Croteau D.L., Mattson M.P., Bohr V.A. (2017). NAD^+^ in Aging: Molecular Mechanisms and Translational Implications. Trends Mol. Med..

[133] Mills K.F., Yoshida S., Stein L.R., Grozio A., Kubota S., Sasaki Y., Redpath P., Migaud M.E., Apte R.S., Uchida K. (2016). Long-Term Administration of Nicotinamide Mononucleotide Mitigates Age-Associated Physiological Decline in Mice. Cell Metab..

[134] Feuz M.B., Meyer-Ficca M.L., Meyer R.G. (2023). Beyond Pellagra-Research Models and Strategies Addressing the Enduring Clinical Relevance of NAD Deficiency in Aging and Disease. Cells.

[135] Ghanem M.S., Caffa I., Monacelli F., Nencioni A. (2024). Inhibitors of NAD^+^ Production in Cancer Treatment: State of the Art and Perspectives. Int. J. Mol. Sci..

[136] Shade C. (2020). The Science Behind NMN-A Stable, Reliable NAD+Activator and Anti-Aging Molecule. Integr. Med..

[137] Revollo J.R., Grimm A.A., Imai S. (2004). The NAD biosynthesis pathway mediated by nicotinamide phosphoribosyltransferase regulates Sir2 activity in mammalian cells. J. Biol. Chem..

[138] Ummarino S., Mozzon M., Zamporlini F., Amici A., Mazzola F., Orsomando G., Ruggieri S., Raffaelli N. (2017). Simultaneous quantitation of nicotinamide riboside, nicotinamide mononucleotide and nicotinamide adenine dinucleotide in milk by a novel enzyme-coupled assay. Food Chem..

[139] You W., Zheng W., Weiss S., Chua K.F., Steegborn C. (2019). Structural basis for the activation and inhibition of Sirtuin 6 by quercetin and its derivatives. Sci. Rep..

[140] Das D., Banerjee A., Mukherjee S., Maji B.K. (2024). Quercetin inhibits NF-kB and JAK/STAT signaling via modulating TLR in thymocytes and splenocytes during MSG-induced immunotoxicity: An in vitro approach. Mol. Biol. Rep..

[141] Akter R., Afrose A., Rahman M.R., Chowdhury R., Nirzhor S.S.R., Khan R.I., Kabir M.T. (2021). A Comprehensive Analysis into the Therapeutic Application of Natural Products as SIRT6 Modulators in Alzheimer’s Disease, Aging, Cancer, Inflammation, and Diabetes. Int. J. Mol. Sci..

[142] Sung M.S., Lee E.G., Jeon H.S., Chae H.J., Park S.J., Lee Y.C., Yoo W.H. (2012). Quercetin inhibits IL-1β-induced proliferation and production of MMPs, COX-2, and PGE2 by rheumatoid synovial fibroblast. Inflammation.

[143] Wang H., Yan Y., Pathak J.L., Hong W., Zeng J., Qian D., Hao B., Li H., Gu J., Jaspers R.T. (2023). Quercetin prevents osteoarthritis progression possibly via regulation of local and systemic inflammatory cascades. J. Cell. Mol. Med..

[144] Hollman P.C., vd Gaag M., Mengelers M.J., van Trijp J.M., de Vries J.H., Katan M.B. (1996). Absorption and disposition kinetics of the dietary antioxidant quercetin in man. Free Radic. Biol. Med..

[145] Galvano F., La Fauci L., Lazzarino G., Fogliano V., Ritieni A., Ciappellano S., Battistini N.C., Tavazzi B., Galvano G. (2004). Cyanidins: Metabolism and biological properties. J. Nutr. Biochem..

[146] Fiorentino F., Mai A., Rotili D. (2021). Emerging Therapeutic Potential of SIRT6 Modulators. J. Med. Chem..

[147] Rahnasto-Rilla M., Tyni J., Huovinen M., Jarho E., Kulikowicz T., Ravichandran S., Bohr V.A., Ferrucci L., Lahtela-Kakkonen M., Moaddel R. (2018). Natural polyphenols as sirtuin 6 modulators. Sci. Rep..

[148] Shozib H.B., Islam M.M., Mahmud S.A.S., Bari M.N., Akter N., Jahan S., Hosen S., Hossain M.N., Nabi A., Siddiquee M.A. (2021). Application of Cyanidin-3-Glucosides as a functional food ingredient in rice-based bakery products. Saudi J. Biol. Sci..

[149] Sato S., Saika A., Koshiyama T., Higashiyama Y., Fukuoka T., Morita T. (2025). Biosynthesis of ergothioneine: Current state, achievements, and perspectives. Appl. Microbiol. Biotechnol..

[150] Cheng J., Keuthan C.J., Esumi N. (2023). The many faces of SIRT6 in the retina and retinal pigment epithelium. Front. Cell Dev. Biol..

[151] Wang Z., Ma J., Miao Z., Sun Y., Dong M., Lin Y., Wu Y., Sun Z. (2023). Ergothioneine inhibits the progression of osteoarthritis via the Sirt6/NF-κB axis both in vitro and in vivo. Int. Immunopharmacol..

[152] Halliwell B., Cheah I.K., Tang R.M.Y. (2018). Ergothioneine—A diet-derived antioxidant with therapeutic potential. FEBS Lett..

[153] Tian X., Thorne J.L., Moore J.B. (2023). Ergothioneine: An underrecognised dietary micronutrient required for healthy ageing?. Br. J. Nutr..

[154] Zhu X., Wen S., Gul H., Xu P., Yang Y., Liao X., Ye Y., Xu Z., Zhang X., Wu L. (2024). Exploring regulatory network of icariin synthesis in Herba Epimedii through integrated omics analysis. Front. Plant Sci..

[155] Gao J., He Y., Shi F., Hou F., Wu X., Yi Y., Zhang Y., Gong Q. (2025). Activation of Sirt6 by icariside II alleviates depressive behaviors in mice with poststroke depression by modulating microbiota-gut-brain axis. J. Adv. Res..

[156] Yang J., Liu Y., He L., Wang Q., Wang L., Yuan T., Xiao Y., Fan Y., Zhang X. (2018). Icariin conjugated hyaluronic acid/collagen hydrogel for osteochondral interface restoration. Acta Biomater..

[157] Hu T., He X.W., Jiang J.G., Xu X.L. (2014). Hydroxytyrosol and its potential therapeutic effects. J. Agric. Food Chem..

[158] Velotti F., Bernini R. (2023). Hydroxytyrosol Interference with Inflammaging via Modulation of Inflammation and Autophagy. Nutrients.

[159] Zhi L.Q., Yao S.X., Liu H.L., Li M., Duan N., Ma J.B. (2018). Hydroxytyrosol inhibits the inflammatory response of osteoarthritis chondrocytes via SIRT6-mediated autophagy. Mol. Med. Rep..

[160] Campus M., Corrias F., Angioni A., Arru N., Sedda P., Addis M., Fiori M., Paba A., Chessa L., Comunian R. (2025). Efficacy of a Native Microbial Starter in Promoting Table Olive Fermentation: An Industrial-Scale Trial at Controlled and Ambient Temperature. Foods.

[161] You W., Rotili D., Li T.M., Kambach C., Meleshin M., Schutkowski M., Chua K.F., Mai A., Steegborn C. (2017). Structural Basis of Sirtuin 6 Activation by Synthetic Small Molecules. Angew. Chem. Int. Ed. Engl..

[162] Jiao F., Zhang Z., Hu H., Zhang Y., Xiong Y. (2022). SIRT6 Activator UBCS039 Inhibits Thioacetamide-Induced Hepatic Injury In Vitro and In Vivo. Front. Pharmacol..

[163] Iachettini S., Trisciuoglio D., Rotili D., Lucidi A., Salvati E., Zizza P., Di Leo L., Del Bufalo D., Ciriolo M.R., Leonetti C. (2018). Pharmacological activation of SIRT6 triggers lethal autophagy in human cancer cells. Cell Death Dis..

[164] Huang Z., Zhao J., Deng W., Chen Y., Shang J., Song K., Zhang L., Wang C., Lu S., Yang X. (2018). Identification of a cellularly active SIRT6 allosteric activator. Nat. Chem. Biol..

[165] Copp M.E., Shine J., Brown H.L., Nimmala K.R., Hansen O.B., Chubinskaya S., Collins J.A., Loeser R.F., Diekman B.O. (2023). Sirtuin 6 activation rescues the age-related decline in DNA damage repair in primary human chondrocytes. Aging.

[166] Shang J., Zhu Z., Chen Y., Song J., Huang Y., Song K., Zhong J., Xu X., Wei J., Wang C. (2020). Small-molecule activating SIRT6 elicits therapeutic effects and synergistically promotes anti-tumor activity of vitamin D_3_ in colorectal cancer. Theranostics.

[167] He T., Shang J., Gao C., Guan X., Chen Y., Zhu L., Zhang L., Zhang C., Zhang J., Pang T. (2021). A novel SIRT6 activator ameliorates neuroinflammation and ischemic brain injury via EZH2/FOXC1 axis. Acta Pharm. Sin. B.

[168] You W., Steegborn C. (2020). Structural Basis for Activation of Human Sirtuin 6 by Fluvastatin. ACS Med. Chem. Lett..

[169] Guo Z., Li P., Ge J., Li H. (2022). SIRT6 in Aging, Metabolism, Inflammation and Cardiovascular Diseases. Aging Dis..

[170] Riegger J., Maurer S., Pulasani S., Brenner R.E. (2022). Simvastatin and fluvastatin attenuate trauma-induced cell death and catabolism in human cartilage. Front. Bioeng. Biotechnol..

